# A library of electrophysiological responses in plants - a model of transversal education and open science

**DOI:** 10.1080/15592324.2024.2310977

**Published:** 2024-03-17

**Authors:** Danae Madariaga, Derek Arro, Catalina Irarrázaval, Alejandro Soto, Felipe Guerra, Angélica Romero, Fabián Ovalle, Elsa Fedrigolli, Thomas DesRosiers, Étienne Serbe-Kamp, Timothy Marzullo

**Affiliations:** a Colegio (High School) Alberto Blest Gana, San Ramón, Santiago, Chile; b Faculty of Medicine, University of Novi Sad, Novi Sad, Serbia; c College of Literature, Science, and Arts, University of Michigan, Ann Arbor, MI, USA; d Hirnkastl, Max Planck Institute for Biological Intelligence, LMU Munich, Munich, Germany; e Research and Development, Backyard Brains, Ann Arbor, MI, USA; f Research and Development, Backyard Brains, Seoul, South Korea

**Keywords:** Plant electrical signals, electrophysiology, environmental stress, plant physiology, comparative physiology, DIY science, open science

## Abstract

Electrophysiology in plants is understudied, and, moreover, an ideal model for student inclusion at all levels of education. Here, we report on an investigation in open science, whereby scientists worked with high school students, faculty, and undergraduates from Chile, Germany, Serbia, South Korea, and the USA. The students recorded the electrophysiological signals of >15 plant species in response to a flame or tactile stimulus applied to the leaves. We observed that approximately 60% of the plants studied showed an electrophysiological response, with a delay of ~ 3-6 s after stimulus presentation. In preliminary conduction velocity experiments, we verified that observed signals are indeed biological in origin, with information transmission speeds of ~ 2–9 mm/s. Such easily replicable experiments can serve to include more investigators and students in contributing to our understanding of plant electrophysiology.

## Introduction

All domains of life, from bacteria to humans, use some form of electrical signaling.^1–5^ Though most commonly associated with the muscle, heart, and nervous system of animals, electrical signals are ubiquitous in life, and, though not as commonly known, also present in plants. The most famous examples of plant electrophysiology deal with rapidly moving plants, such as the venus flytrap (*Dionaea muscipula*)^6–8^ and the sensitive mimosa (*Mimosa pudica*)^9–12^ that generate action potentials when touched. Electrical signaling, however, is also documented in plants that do not necessarily have rapid movement behavior, such as the tomato (*Solanum lycopersicum*),^13^ sundew (*Drosera),*^14^ arabidopsis *(Arabidopsis thaliana)*,^15^ corn (*Zea mays)*,^16^ avocado (*Persea americana*), and plum (*Prunus domestica*),^17^ among others.

Plant electrophysiology is understudied and ripe for further experimentation. For example, a PubMed search in 2023 (https://pubmed.ncbi.nlm.nih.gov/) revealed 68 papers published on plant electrophysiology, 986 on animal electrophysiology, and 2280 on human electrophysiology. Such publication rate difference is curious given that plant electrophysiology experiments are relatively inexpensive and lack regulatory hurdles. Cataloging plant electrophysiology responses is of value to the scientific community, and such experiments are uniquely positioned for the high school and undergraduate teaching environment. The hypothesis of the work presented here is that electrophysiological responses are common in plants; thus we emphasize this by recording from a wide variety of plants. Moreover, plant electrophysiology allows for greater inclusion of young scientists in the process of contributing to the scientific literature.

There is the intriguing and provocative hypothesis that if plants have electrical signals similar to properties of nervous systems, therefore perhaps plants have properties similar to classically considered intelligent lifeforms (animals).^18^ Inter-plant communication has been documented in underground root networks,^19^ as well as inter-plant hormone release communication with volatile organic compounds (VOCs).^20,21^ Traditional measures of intelligent behavior have emphasized the human time scale over the plant time scale, hence the continuing debate.^22^ Other scientists have emphasized that intelligence can be viewed as “adaptively variable behavior within the lifetime of the individual”.^23,24^ That plants can respond to stimuli and change their behaviors has been documented many times (for review, see).^25^ For example, plants may be capable of adaptation and classical conditioning.^26,27^ Enabling plant electrophysiological experiments to be accessible at the high school/undergraduate level gives students the ability to contribute to areas of active debate such as learning and information processing in plants,^26–28^ and other concepts such as attention.^29^

Action potentials vary in duration across the domains of life. Mammals tend to have fast communication systems, with action potentials on the order of ms, and conduction velocities on the order of 3–80 m/s, while plants are 1000–15,000× slower, in the mm-cm/s range.^30^ A neuron action potential is typically 1 ms long, a muscle action potential is 3 ms long, and a heart action potential is typically 200 ms long.^31^ The mV values of such action potentials are ~-70-80 mV to +50 mV. However, the algae cell *Chara*, for example, has an action potential duration of multiple seconds and mV values of −180 mV to −60 mV (Johnson et al. 2002). The tomato plant wound potential is in the 10 mV order range with time scales of seconds as well.^32^

Bacteria, notably, use electrical signaling (an action potential) across the cell membrane to begin the formation of spores, changing electrical potential over a number of hours.^4^ Action potentials can be present in fungus, with spikes ranging from µV to mV and potentials lasting hours long.^1^ Slime molds can generate impulses in the seconds range.^33^

In all systems, the generation of electrical signals is due to the movement of ions inside and outside of membranes, but the propagation of signals is different across domains. In animal systems, the propagation of electrical systems in the heart, brain, and muscles is through synaptic transmission. In single-celled organisms such as single cell fungus and bacteria, the propagation of the action potential is limited to the cell *in situ.*

In plants, generally, there are action potentials, variable potentials, long potentials, and slow potentials – the function of each being of continued debate.^34^ However, it can be stated that an organism’s inability to generate an electrical signal can be considered a sign of organism death.^35^ In the human world, for example, a human is not considered dead until they register a flat electroencephalogram.^36^

The propagation of electrical signals in plants remains an area of active investigation, but is hypothesized to travel via the plant’s vascular system of xylem and phloem.^32,37–40^ Xylem is a structure that transports water and minerals up the plant, while phloem transports bidirectionally, up and down, including the sap from photosynthesis.^41^ However, non-vascular plants such as moss are also capable of generating electrical potentials,^42^ which merits further investigation into the anatomical basis of electrical signaling in plants. In initiation of such studies, we hereby report the inauguration of work to more systematically understand electrical signaling in a variety of commonly available vascular plants.

**Figure 1.**
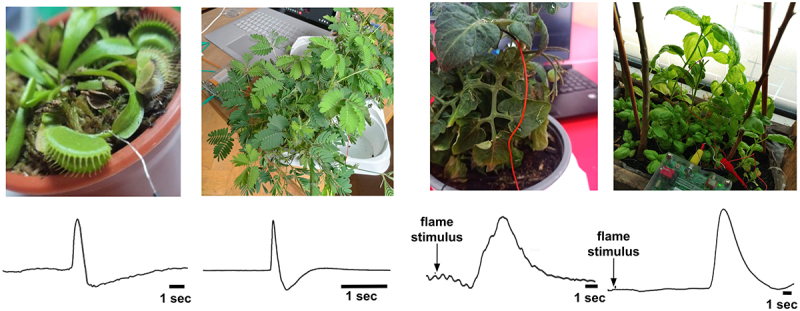
Exemplary single trace data from venus flytrap (*Dionaea muscipula*), sensitive mimosa (*Mimosa pudica*), tomato (*Solanum lycopersicum*), and basil (*Ocimum basilicum*). The venus and mimosa plants received a tactile stimulus, while the tomato and basil plants received a flame stimulus.

With this goal in mind, the work reported here consisted of five high school students (DM, DA, CI, AS, FG), two high school faculty (AR, FO), two undergraduate students (EF, TD), and two scientists (ESK, TM) working together collecting plant electrophysiology data during the 2022–2023 school year. In the specific experiments described in this paper, we recorded from the branches of 16 different plant species in response to flame or tactile stimuli, with ~ 60% of the plants showing electrophysiological responses to the stimuli (examples - Figure 1). Plant electrophysiology is positioned to allow more lateral movement between all levels of education from high schools to advanced research institutes.

## Materials and methods

**Figure 2.**
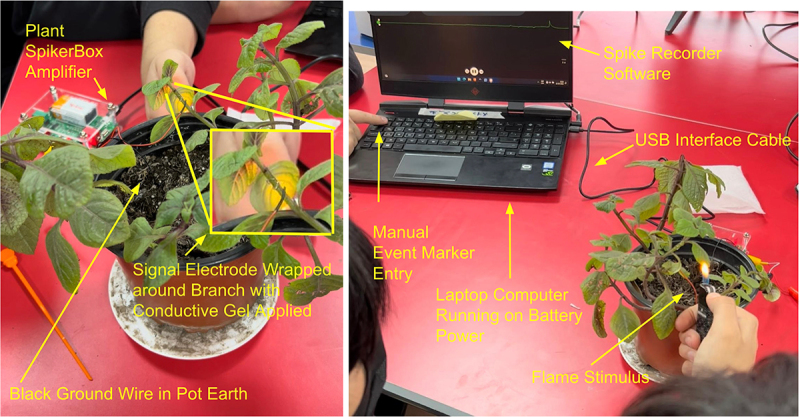
Experimental setup using Argentinian dollar (*Plectranthus purpuratus*) as an example. Note student manually marking the point of flame stimulus presentation on the recording software via numerical keystroke.

The electrodes used to record from the plants were 127 µm bare silver wire (A-M systems 781,500) wrapped in a spiral 1–3 times around the plant branches, approximately 2–4 cm distal to the leaf being studied. Conductive electrode gel (signa gel, Parker Laboratories, part number #15–60) was applied to the spiral wire to improve signal stability. The ground wire consisted of a standard map pin wire placed into the moist ground of the potted plant (Figure 2). The signals were amplified with a Plant SpikerBox (gain 72×, 0.07–8.8 Hz bandpass filter) (Backyard Brains) and sent via a USB serial interface (10 kHz sampling rate) to a Hewlett Packard, Dell, or Windows Surface laptop computer running on battery power. The software was recorded with the Backyard Brains “Spike Recorder’’ program as .wav files with event markers stored as .txt files for time logging when the stimulus was applied and removed. Event markers were entered manually by the students during the experiments by pressing the “1” key on the keyboard for when the stimulus was applied and “2” when the stimulus was removed. After each experiment, data was manually uploaded to a Google Drive cloud storage site accessible to all authors.

Experiments were done 80% remotely, with the high school students and faculty (Chile) logging into a Google Hangouts video link, and the two scientists (ESK, Germany, TM, South Korea) guiding the experiments the high school students performed. The last month of the class in Chile was done presentially, with TM visiting the high school for the final classes and ESK logging in remotely. The experiments the undergraduates (EF, TD) did were done during a four-week summer fellowship in Belgrade, Serbia with the scientists ESK and TM physically present. Effective use of online tools (Google Hangouts, Google Colab, and Google Drive) was crucial for the success of this project.

The plants examined in detail (more than 3 experiments over different days) were 1- Araucaria (*Araucaria araucana*), 2- Argentinian Dollar (*Plectranthus purpuratus*), 3- Basil (*Ocimum basilicum*), 4- an unidentified fern species (Polypodiopsida), 5- Ivy (*Hedera*), 6- Lemon Balm (*Melissa officinalis*), 7- Mint (*Mentha spicata*), 8- Oregano (*Origanum vulgare*), 9- Papyrus (*Cyperus papyrus*), 10- Radiators (*Peperomia*), 11- Rosemary (*Salvia rosmarinus*), 12- Ruda (*Ruta graveolens*), 13- Sensitive Mimosa (*Mimosa pudica*), 14- Sundew (*Drosera capensis*), 15- Tomato (*Solanum lycopersicum*), and 16- Venus Flytrap (*Dionaea muscipula*), all purchased from various local suppliers (Table 1, Supplementary Figure S1). For the venus flytraps, we used tactile stimulation of the trap trigger hairs with a plastic probe as the stimulus. For the sensitive mimosa, we used a tactile stimulus of the leaves with a plastic probe. For all other plants, we used flame applied to the apical tip of a selected leaf closest to the recording electrode for 2–4 s as the stimulus. The flame stimulus consisted of flexible long-neck butane lighters (Ronson) carefully positioned to minimize any movement of the plant leaves and branches during experiments to reduce electrical artifacts. The flame stimulus was chosen as a stimulus to minimize any electrical noise caused by movement of the leaves or contact with metal scissors (cutting stimulus). Typical appearance of the leaf after burning was a charring around the leaf apical tip. All plants studied had a minimum of 2 members of the species studied (such as fern and papyrus), while others had up to 9 (such as mint and sundew) (Table 1).

**Table 1.** Summary of all plants experimented on.

Common Name	Latin Name	Electrical Response	#Recordings	#Plants
Araucaria	*Araucaria araucana*	No	11	3
Argentinian Dollar	*Plectranthus purpuratus*	Yes	30	3
Basil	*Ocimum basilicum*	Yes	42	6
Fern	Polypodiopsida	No	7	2
Ivy	*Hedera*	No	10	3
Lemon Balm	*Melissa officinalis*	No	11	4
Mint	*Mentha spicata*	Yes	28	16
Oregano	*Origanum vulgare*	No	5	5
Papyrus	*Cyperus papyrus*	No	10	2
Radiators	*Peperomia*	No	8	2
Rosemary	*Salvia rosmarinus*	Yes	37	8
Ruda	*Ruta graveolens*	Yes	42	5
Sensitive Mimosa	*Mimosa pudica*	Yes	40	6
Sundew	*Drosera capensis*	Yes	20	9
Tomato	*Solanum lycopersicum*	Yes	27	10
Venus Flytrap	*Dionaea muscipula*	Yes	70	5
		Total	398	89

It is important to highlight the difference between tactile stimulation and flame stimulation. We used tactile stimulation for well-characterized systems like the venus flytrap and the sensitive mimosa, but we used flame-induced wound responses to investigate electrical signaling in non-rapid movement plants. Tactile stimulation in non-rapid movement plants often results in movement artifacts, so to reduce this risk, we elected to use heating responses with a flame to demonstrate electrical potential presence in such plants.

In some instances, the electrode technique varied. For the venus flytraps, the recording electrode consisted of a plastic stake placed into the ground with a spiral wrap of silver wire around the tip, and the spiral tip of silver lain against the side of the trap, with conductive gel applied to the silver spiral. For some of the araucaria, radiator, and papyrus experiments, the ground electrode consisted of a stainless steel map pin needle placed directly into the stem instead of the moist soil. Experiments were performed approximately once a week from late August 2022 to March 2023 during the approximate spring and summer seasons in Santiago, Chile, and during a four-week 2023 summer fellowship with undergraduate students in Belgrade, Serbia.

To analyze the data, we used custom Google Colab notebooks that could access our recordings shared in a Google Drive. The software routines were written in Python, and code base examples can be seen in the “Online resources” links at the bottom of this manuscript. Our “spikertools” library analyzed the plant electrophysiological responses before and after the flame/tactile stimulus was applied using the embedded event markers. All recordings were normalized for signal amplitude, peak-aligned, and sign inverted when the peak response was negative.

The sign inversion was implemented solely for normalization purposes, specifically applied when the extremum of a recorded signal was a negative minimum. This step was essential to align the signals for comparative analysis. It is important to note that this inversion was not intended to assert the correct polarity of the signals. Our primary objective was to investigate the presence or absence of wound potentials, rather than to definitively determine their true polarity.

**Figure 3.**
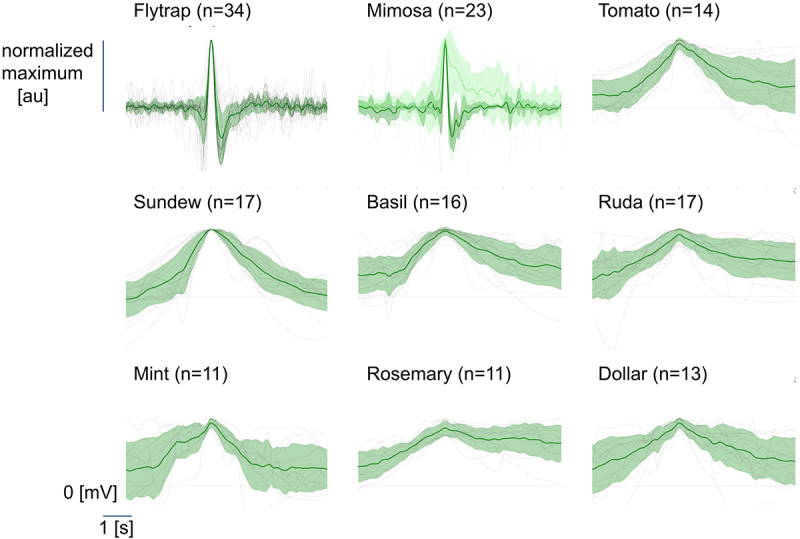
Library of electrical discharges from 9 plants. Robust flytrap (*n* = 34) and mimosa (*n* = 23) action potentials elicited by tactile stimuli. Mimosa recordings showed fast (*n* = 14, green) and slow (*n* = 9, light green) signals. Other plant recordings show putative wound potentials obtained through a flame stimulus. Depending on the stimulus, different data analysis was performed (see methods). Individual gray traces depict single experiments and green lines show the overall mean with the standard deviation as shaded areas. Horizontal dashed lines at 0 for reference after baseline subtraction. Vertical bar depicts the maximum of the normalized responses. au = arbitrary units.

To determine general response patterns (see Supplementary Figure S1) we calculated the mean over all individual recordings where we detected at least 10 peaks after artifact rejection (Figure 3 - the green line represents the mean with standard deviation shades, and the gray traces depict individual recordings). Plants were considered to have responses to the stimuli if the recorded signal deviated more than 75% outside the signal range prior to the stimuli.

In electrophysiological studies, especially in plants, distinguishing between true signal responses and background noise is a significant challenge. Lower thresholds like 50% or 25% might increase the sensitivity of detection but also substantially elevate the risk of false positives, where random fluctuations or noise could be misinterpreted as legitimate responses. Given the subtlety of plant responses compared to more excitable tissues like animal neurons, it is crucial to establish a threshold that provides a good balance between sensitivity and specificity.

Our decision to set the threshold at 75% was reinforced by our findings in detecting action potentials in mimosas and venus flytraps, which we used as a ground truth for our analysis algorithm. In our preliminary experiments with these plants, we observed that a 75% deviation threshold reliably differentiated between actual physiological responses and background noise or movement artifacts. This threshold was not only effective in minimizing the likelihood of false positives but also consistent with the response patterns observed in these species, which are well-documented in plant electrophysiology research.^6–12^

Automated data processing enabled the selection of the data for anticipated signals. We filtered out traces that 1) had spikes faster than 500 ms (touching artifacts of the recording wire) 2) were exceeding the recording range (clipping artifacts) 3) were exhibiting spikes that occurred over an elevated baseline level of 75% (normalization artifacts). “Online resources” at the end of this manuscript lists the permalinks to the data analysis code. This analysis allows us to add more recordings to our dataset, but also to extend the automated signal detection for new plant species.

**Figure 4.**
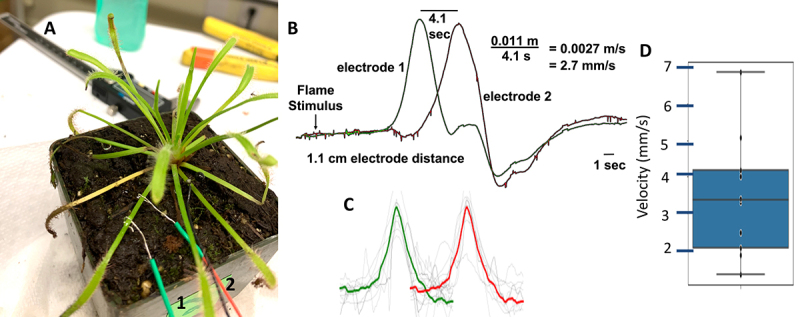
Conduction velocity measurements in sundew *(Drosera capensis)* using a flame stimulus. a. Experimental setup with two signal electrodes along one branch. b. Exemplary recording. c. Data and average signal from 11 different recordings on 9 plants from two channels (green and red). d. Scatterplot of same 11 recordings. Average conduction velocity is 3.2 mm/s.

In final experiments, we measured the conduction velocities of electrical impulse transmission in the tomato (*Solanum*), sundew (*Drosera*), and sensitive mimosa (*Mimosa*) plants (Figure 4). To record the conduction velocities, we used a 2-channel prototype amplifier that consisted of two custom amplifier shields (bandpass 0.2–130 Hz, gain ~ 55×) placed on top of an Arduino Uno for signal acquisition. Two wires (channel 1, channel 2) were placed with conductive gel approximately 1–2 cm apart on a branch and a ground pin placed in the ground (sundew/mimosa) or in the stem (tomato). For the tomato and sundew measurements, a flame stimulus was used. For the sensitive mimosas, a tactile stimulus was used (a strike on the leaves with a plastic probe). The distance between the two signal wires on the branch and the time difference between the two peaks on the two channels was used to calculate conduction velocity.

## Results

Figure 1 shows exemplary data (individual traces) from a venus flytrap, sensitive mimosa, tomato, and basil plant. The venus flytrap and mimosa show responses to tactile stimuli, while the tomato and basil recordings show responses to flame stimuli. Note the obvious change in signal when the stimulus was applied.

Figure 2 depicts the standard experimental set-up used by the students, which they learned rapidly to operate (within one session). Unless otherwise noted in the methods above, a silver wire signal electrode was wrapped around the branch, conductive gel was applied, and a map pin ground wire was placed in the moist earth of the potted plant. The signal was amplified with our custom Backyard Brains “Plant SpikerBox” amplifier, and the electrophysiological responses were recorded using our “Spike Recorder” software.

Table 1 shows the list of all 16 plants studied with a total number of 398 recordings obtained over a period of on average one session per week during 7 months spread out over one school year (spring-summer – Chile; summer – Serbia).

Figure 3 demonstrates the data from the 9 of 16 (56%) plants that demonstrated a putative response to the flame or tactile stimulus. The plants that showed responses include *Ocimum* (basil), *Dionaea* (venus flytrap), *Ruta* (ruda) , *Drosera* (sundew), *Solanum* (tomato), *Mimosa* (mimosa), *Mentha* (mint), *Salvia* (rosemary), and *Plectranthus* (Argentinian dollar). If the amplitude exceeded 75% of the average signal prior to the stimulus, the recording was considered a putative response. Of note is that the responses can be seen in plants (7 of 9) that do not necessarily exhibit rapid movement in their behaviors as well as in common household plants that are available for purchase globally – such as the *Solanum* (tomato), *Ocimum* (basil), and *Mentha* (mint).

Figure 4 shows experimental results on conduction velocity measurements in *Drosera* (sundew) in response to a flame stimulus. Conduction velocities were on the order of 2–9 mm/sec. Preliminary conduction velocity experiments were also done in tomato plants, with an observed conduction velocity of ~ 9 mm/s in response to a flame stimulus, and in the sensitive mimosa a conduction velocity of ~ 8 mm/s in response to tactile stimuli (data not shown).

## Discussion

Experiments in plant electrophysiology allow students and faculty to appreciate plants as dynamic systems with metabolic cycles occurring in real time, even though (to humans), plants can appear as static organisms. Outside of exotic plants like sensitive mimosas and venus flytraps (and the very reason they are exotic is because they move), the lack of perceived movement (a powerful cue for animal visual systems^43^) can make plants seem less alive and present,^44,45^ a term called “plant blindness”^46^ or a growing alternative name “plant awareness disparity”.^47^ Such experiments are open-ended and readily reproducible, facilitating result verification by scientists and students globally. Moreover, our online data repository welcomes additional observations, fostering collaborative contributions to the field.

In this work, we demonstrated and verified that many plants (not necessarily rapid-movement plants) have robust electrical signals. Studying 16 different species is a high number from an individual experiment point of view, but not considering the high biodiversity of plants in the world. The number of plant species is estimated to be 400,000,^48^ which seems oddly small and potentially underreported, as there are more documented animal species, mostly due to insects (>900,000 insect species).^49^ Our hope is that electrophysiology in many diverse plant species becomes commonplace in educational institutions globally, increasing our understanding of information transmission in plants, a subject we have anecdotally found that teachers and students find fascinating.

Though not systematically studied, we also examined other plant species in one-off experiments, such as clover (*Trifolium*), chile (*Capsicum*), touch-me-not (*Impatiens*), rose (*Rosa)*, and carrot (*Daucus)*. We observed signals while stimulating through cutting or flaming different parts of the plants (leaves, fruits, stem). The chile, rose, and touch-me-not signals are compelling, but require an in-depth investigation. Studying the electrophysiological systems of plants valuable for the global food supply (such as tomato, chile, and carrot) can have social implications – could electrophysiological monitoring systems for plants help in greenhouse agriculture for automated health detection?^13^

Plant electrophysiology is paradoxically both easier and harder than animal electrophysiology. It is easier as the experiments are relatively simple to set up, but without a behavior observable on a human time scale, interpreting whether observed electrophysiological data is biologically relevant and real remains the primary challenge. Disregarding noise sources systematically is the main experimental difficulty for novices. By building easily understandable artifact rejection routines into our analysis script, we can reduce bias in data visualization of our recordings that could potentially be caused through “hand sorting” (see Supplementary Figure S1 for a representation of detected peaks in all data recorded without artifact rejection applied).

In all plants aside from the venus flytrap and sensitive mimosa, the responses were very variable. For newly investigated plants that exhibited potential wound responses, we only detected them in ~ 30% of all total recordings. The variability is most likely linked to the health and physiological state of the individual plants used. For example, in mint plants, sometimes, at the same time of day, in the same environmental conditions, one plant would show robust responses to the flame stimuli, while the next plant investigated of the same species would not reveal any responses. Thus we make the statement “putative” to state that though the analysis automatically went through our algorithm, determining the origin of such variability is obviously of interest for future experiments. We found the non-rapid movement plants with the most “robust” and “reliable” wound responses were the basil and tomato plants (Figure 1), a pleasant surprise given how easy it is to purchase these plants in all five countries involved in this study. Interestingly, to our knowledge, we were the first ones to describe the electric potentials of wound responses in the basil plant, where secondary effects of wounds are well studied describing phenolic compound production, VOC release, and hormonal responses.^50^

Our plant amplifier equipment, being AC amplifiers and not DC amplifiers, and not doing whole cell recordings, do not give us a true indication of mV value at the moment. We can detect the time a potential occurred, the width of the potential, and the extracellular potential shape, but we cannot as yet report the true mV values. Future protocols will develop a calibration scheme and will also involve whole cell recording, beginning by replicating experiments in the multicellular algae *Chara coralina*, a member of the Characeae family.^3^

A major innovation of this paper is the inclusion of young scientists in the collection of the data, the use of inexpensive, accessible equipment, and the high number of recordings (~ 400 recording sessions). As all of our data is on an online repository (see “Online resources” at the bottom of this manuscript), whereby other investigators (professional scientists, students, and citizen scientists), can add their own experimental data, we hope to develop a large “citizen science” library of plant electrophysiological response data that scientists around the world can access and analyze.

It is a non-trivial part of this work that high school students did the majority of the data collection, showing the advantages that plant electrophysiology has for introducing young students to the scientific process of data collection, data analysis, and manuscript writing, something most aspiring scientists are only exposed to in late undergraduate education at the earliest, if lucky. The participation of professional scientists on a consistent, long-term basis with young researchers is critical for such initiatives. Young students can record data and discuss results, but analyzing the literature and the technical writing is the biggest challenge, which requires active oversight and collaboration by scientist colleagues.

We recorded principally angiosperms (flowering plants); the only gymnosperms were young araucaria trees, called Pehuén and considered holy by the Mapuche, and the only Polypodiopsida was one unidentified fern species. We did not try to record from non-vascular plants such as mosses, though that would be an interesting future direction.

As we observed 56% of the plants we studied had electrical responses to our stimuli, does this mean that the other 44% did not have electrical responses, or merely that we failed to observe them? The latter seems more likely given how ubiquitous electrical signaling is in biology. Effects on the responsiveness of the plant may include the dormancy state of the plants, health of the plant – whether the plant has already been attacked by insects (we anecdotally observed plants with herbivore wounds did not manifest electrophysiology wound responses, but did not systematically study this), moisture level of the soil (ensuring a stable ground), among other factors. Another potential source of noise can be the signal interface, as most of our plants with observed responses were relatively small with slender, pliable green stems (<1-m tall), whereas other plants such as the papyrus and rosemary had drier stems and branches.

The electrode interface varied based on our previous exploratory experiments with tomato, venus flytrap, sundew, and sensitive mimosa. With the venus flytrap, being a mature experiment we have demonstrated for 8 years in public, we have found that the plastic stake in the ground with a silver coil at the tip resting along the trap’s side is the simplest for users to replicate. However, a spiral wire wrapped around the leaf branch closest to the trap, similar to our protocol with all the other plants we studied, also reveals stable recordings (Figure 1, left). With regard to the ground pin sometimes being placed in the stem versus being placed in the ground, due to the highly variable nature of plant electrophysiology, sometimes the students placed a ground wire in the stem (per scientists ESK and TM’s advice, in an attempt to stabilize recordings if recordings appeared noisy). It is a technique scientists ESK and TM have sometimes used in other experiments not reported here, but generally try to avoid to evoke additional wound responses in the plant.

With large plants like trees, would a simple spiral electrode of silver covered with conductive gel be sufficient for recording a signal? For example, in the araucaria plant, the leaves and branches are very tough and waxy. Was a lack of observed response due to an improper interface with the plant, or that the plant lacks electrical signals? Testing with various different electrode designs would bear this out. Given how ubiquitous electrical signals are across all domains of life, such a lack of electrical response seems unlikely in the plants where we didn’t observe a response. It is more likely due to improper interface design.

In preliminary conduction velocity studies, we observed that plant conduction velocity is on the order of ~ 2-9 mm/s. This is of note, as sensory nerve neural conduction velocities in humans is ~ 50–80 m/s^51^ and in the earthworm, 10–20 m/s.^52^ Thus, plants are transmitting information throughout their vascular structures on an order of 1000–15,000× slower than animals’ neural systems.

The questions that emerge from this work are: 1) How are these electrophysiological signals generated in the plant?, and 2) How far do these electrical signals propagate? Future experiments will involve systematically measuring conduction velocity across the plants studied in this work to visualize the propagation paths and anatomical structures (Figure 4). We are now investigating this in the same plants from this study with an undergraduate student from the University of Santiago, Chile. An additional question is: what is the function of such signals?^30^ Plants face the “stuck in place” problem, thus they have developed defenses against attack, most notably in the willow tree, tomato, tobacco, and sensitive mimosa.^53^

Most studies on plant physiology rely on model organisms like *Arabidopsis* and tomato, for obvious reasons such as the depth of literature, the understanding as a model organism, and the rapid growth cycle. But plants are ubiquitous and accessible across the world, and thus there is the potential for rapid innovation in our understanding of life systems by citizen scientists.

It is our hope that in the future more and more scientists contribute to a database of plant electrophysiology, and we improve our enlightenment of one of the least appreciated, though no less marvelous, domains of life. Our work here, with high school students collecting the majority of the data, speaks to the universal possibility of increased public participation from all realms in the scientific process of understanding biological information-processing systems.

## Online resources

To generate the figures Figure 3 and Supplementary Figure S1, we used the following code base: t.ly/dJBGc

To generate Figure 4 (conduction velocity in *Drosera*), we used the following code base: t.ly/HRdQA

The online data from all recordings is stored in the following library: t.ly/imDXR

New data can be sent also through Google Forms: t.ly/LbTTI

## Supplementary Material

Supplemental Material

SupplementalFigure1.jpeg
