# Supplementary material for: A library of electrophysiological responses in plants - a model of transversal education and open science
Source: Plant Signal Behav. 2024 Mar 17;19(1):2310977. doi: 10.1080/15592324.2024.2310977 (PMC10950275; doi:10.1080/15592324.2024.2310977)
Supplement: Supplemental Material [file KPSB_A_2310977_SM1689.zip › Plant E.Phys. Source Data /Mentha/Oct 30 2022 mint conduction velocity/Mint Conduction Velocity Notes.docx]

I - 13 mm

II - 17 mm

III - 26 mm
